# Continuity of care between dyslipidemia patients and multiple providers: A cohort study

**DOI:** 10.1371/journal.pone.0300745

**Published:** 2024-05-02

**Authors:** Eunyoung Choi, Juhee Lee, Eunjung Choo, Eun Jin Jang, Iyn-Hyang Lee

**Affiliations:** 1 College of Pharmacy, Yeungnam University, Gyeongsan, Republic of Korea; 2 Department of Pharmacy, Ulsan University Hospital, Ulsan, Republic of Korea; 3 Department of Statistics and Probability, Michigan State University, East Lansing, MI, United States of America; 4 College of Pharmacy, Ajou University, Suwon, Republic of Korea; 5 Department of Informational Statistics, Andong National University, Andong, Republic of Korea; 6 Department of Health Sciences, University of York, York, United Kingdom; University of Porto, Faculty of Medicine, PORTUGAL

## Abstract

**Objective:**

This study aimed to investigate the impacts of continuity of care (COC) between patients and multiple providers, i.e., doctors and community pharmacists, on clinical and economic outcomes.

**Methods:**

This is a retrospective cohort study and analyzed Korean national claims data for ambulatory care setting between 2007 and 2018. Patients with dyslipidemia newly diagnosed in 2008 were identified. COC between providers and patients was computed using the continuity of care index (COCI). Based on COCIs, the study patients were allocated to four groups: H^M^/H^P^, H^M^/L^P^, L^M^/H^P^, and L^M^/L^P^. Each symbol represents H for high, L for low, M for doctor, and P for pharmacist. The primary study outcome was the incidence of atherosclerotic cardiovascular disease (ASCVD).

**Results:**

126,710 patients were included. Percentages of patients in the four study groups were as follows: H^M^/H^P^ 35%, H^M^/L^P^ 19%, L^M^/H^P^ 12%, and L^M^/L^P^ 34%. During the seven-year outcome period, 8,337 patients (6.6%) developed an ASCVD, and percentages in the study groups were as follows; H^M^/H^P^ 6.2%, H^M^/L^P^ 6.3%, L^M^/H^P^ 6.8%, and L^M^/L^P^ 7.1%. After adjusting for confounding covariates, only the L^M^/L^P^ group had a significantly higher risk of ASCVD than the reference group, H^M^/H^P^ (aHR = 1.16 [95% confidence interval = 1.10~1.22]). The risk of inappropriate medication adherence gradually increased 1.03-fold in the H^M^/L^P^ group, 1.67-fold in the L^M^/H^P^, and 2.26-fold in the L^M^/L^P^ group versus the H^M^/H^P^ group after adjusting for covariates. Disease-related costs were lower in the H^M^/H^P^ and L^M^/H^P^ groups.

**Conclusions:**

The study shows that patients with high relational care continuity with doctors and pharmacists achieved better clinical results and utilized health care less, resulting in reduced expenses. Further exploration for the group that exhibits an ongoing relationship solely with pharmacists is warranted.

## Introduction

Management of illnesses is frequently neglected by patients [1,2], and poor management of chronic illnesses is a major issue that undermines the effective delivery of healthcare [3]. Dyslipidemia must be managed for a long time and could be sometimes poorly managed [4–6]. Poor management of dyslipidemia is correlated with a high risk of atherosclerotic cardiovascular diseases (ASCVDs) [7,8], and has been linked to higher mortality rates [9]. Thus, proper management of dyslipidemia is considered a central component of ASCVD risk management [4,10]. The factors that militate against dyslipidemia treatment are a lack of perceived symptoms [5] and treatment misconception [6]. The most recent studies reported that continuous provision of care in patients with dyslipidemia could reduce the incidence of ASCVDs [6,11].

Recently, a growing number of studies have explored the role of continuity of care (COC) in patients with chronic diseases [12–15]. COC is widely accepted as a connected and coherent series of healthcare events consistent with an individual’s needs, which are categorized as; informational, management, and relational COC [16]. Informational continuity can be understood to involve sharing medical histories among past and present providers, management continuity involves the coordination and integration of care in accordance with a care plan shared agreed upon by different providers, and relational continuity involves lasting therapeutic relationships between patients and their providers [16]. Existing literature supports the notion that high relational continuity lowers the risk of premature mortality among those with asthma or chronic obstructive pulmonary disease [17], emergency visits by elderly patients [18], hospitalizations of patients with chronic kidney disease [19], and the medical expenses of those with a chronic disease [20].

The majority of studies on this topic have focused on issues regarding relational continuity between patients and doctors. One systematic review reported that high COC between patients and community pharmacists might increase safe drug use, but other clinical or economic consequences were seldom identified [21]. Indeed, the impacts of COC between patients and multiple providers have rarely been investigated. In this study, we explored relational COC levels with doctors and community pharmacists in patients with dyslipidemia to investigate the impacts of COC on clinical and economic outcomes. We hypothesized that patients with high relational care continuity with their doctor and pharmacist achieve better clinical results, and incur lower treatment-related costs.

## Materials and methods

This study was performed in accordance with the Declaration of Helsinki and was reviewed beforehand by the Institutional Review Board of Yeungnam University (IRB no. 201804004002). The requirement for participant consent was waived because the study was conducted using anonymous claims data provided for research purposes by Korean National Health Insurance Service (KNHIS). This retrospective cohort study was conducted in accordance with the Strengthening the Reporting of Observational Studies in Epidemiology (STROBE) guideline [22].

### Data sources

We analyzed anonymized national insurance claims data between 2006 and 2018 provided by KNHIS. KNHIS claims data contain de-identified patient socio-demographic information, diagnoses, all medical services provided and medications dispensed, and death records [23]. Data analysis was performed in January 2020~ March 2021.

### Study timeframe

The study period was from 2007 to 2018 which illustrated in S1 Fig. Index dates were defined as the earliest dates of the claims of patients diagnosed with dyslipidemia in 2008. The history period covered one year before the index dates, the exposure period included the three years following the index dates, and the outcome period encompassed the subsequent seven years. Exposure and outcome periods were separated because inadequate temporal relationship could overestimate outcomes [24]. Each patient was followed from the end of the exposure period until a diagnosis of ASCVD, death, or the end of data collection.

### Study population

The study involved outpatients newly diagnosed with dyslipidemia in 2008. Confirmation of dyslipidemia was based on the International Classification of Disease 10th revision (ICD-10) classification, encompassing codes E78.0 to E78.9. To ensure the inclusion of only recently diagnosed patients, individuals diagnosed with dyslipidemia during the history period were excluded. Patients who made at least two ambulatory doctor visits and at least two pharmacy visits during the index year and at least four ambulatory doctor visits and at least four pharmacy visits during the exposure period were included in the study [24–26]. Since the implementation of the separation of prescribing and dispensing in 2000, outpatients in Korea can obtain their prescribed medications from community pharmacies. Therefore, in this study, a ’pharmacy’ specifically refers to a community pharmacy.

Patients were excluded if they had pre-existing cardiovascular conditions including hypertensive disease, ischemic heart disease, cerebrovascular disease or a related syndrome, diabetes mellitus with circulatory complications, or cancer from January 1, 2006 to the index dates, because of possible relations between these diseases with the study outcome or health care utilization. In addition, we also excluded those that had received a diagnosis of myocardial infarction, stable or unstable angina, ischemic stroke, or a transient ischemic attack, and those that died during the exposure period. All ICD-10 codes used for data analysis are reported in S1 Table.

### Measuring continuity

Continuity of care is broadly conceptualized to the extent to which one patient’s visits are concentrated among providers and was measured using the Bice & Boxerman Continuity of Care Index (COCI) [20,25,27]. COCI is recommended for use on situation of South Korea where patients are largely free to contact different doctors and pharmacists due to a weak gatekeeper role of primary care [28]. The formula to calculate Bice & Boxerman COCI is reported in the supporting information (S1 File).

### Configuration of continuity cohort

Interim analyses were performed to determine the COCI distributions of doctor and pharmacist visits during the exposure period, and showed that patients with a COCI of ≥ 0.8 accounted for 54.4% of doctor visits and 46.9% of pharmacist visits (S2 Fig). As far as we are aware, there is no generally used cut-off value for high and low COCI. The COCI would be 0.8, if a patient made ten visits that included nine visits to the same provider. From a practical perspective, a patient who made 9 of 10 visits to the same provider was considered to have a high continuity relationship with that provider. With this regard, a COCI of ≥ 0.8 during the exposure period was defined as high COC. The study patients were allocated based on high or low doctor and pharmacist COCIs to four groups: H^M^/H^P^, H^M^/L^P^, L^M^/H^P^, and L^M^/L^P^. Each symbol represents H for high, L for low, M for doctor, and P for pharmacist.

### Outcome measures

The primary outcome was the incidence of ASCVD during the outcome period. We defined ASCVD to include myocardial infarction, stable or unstable angina, ischemic stroke, or transient ischemic attack based on previous research [7,29]. The disease was confirmed as follows: (1) a diagnosis of myocardial infarction treated by percutaneous coronary intervention (PCI) or coronary artery bypass graft surgery (CABG); (2) a diagnosis of stable or unstable angina treated by PCI or CABG; (3) a diagnosis of ischemic stroke assessed by brain imaging (CT or MRI); and (4) a diagnosis of transient ischemic attack assessed by brain imaging (CT or MRI) (S1 Table). Secondary outcomes included health services utilization, disease-related medical costs, and medication adherence. Health services utilization was assessed using the number of patients hospitalized or visiting emergency departments (EDs) due to ASCVD. Related medical costs included expenses attributable to dyslipidemia and ASCVD, which were sum of costs in claims identified by the ICD-10 diagnosis codes (S1 Table). Medication adherence of antihyperlipidemic agents was assessed using medication possession ratios (MPRs). We defined an MPR of < 0.8 inadequate medication adherence [30].

### Covariates

Covariates included individual characteristics such as sex and age. Insurance contributions were classified as high, moderate, and low, and used as a proxy of patients’ economic circumstances. In KNHI, there are two types of health programs, viz NHI and MedAid. About 97% of the population is covered by NHI and 3% by MedAid [31]. Most of the Korean population is covered by a mandatory National Health Insurance scheme, complemented by a Medical Aid system that provides more comprehensive coverage to vulnerable populations, such as low-income households. Locations of residences at the index dates were classified into large urban, small urban, and rural areas. In parallel with Korean administrative divisions, we defined metropolitan cities with a population density exceeding one million as large urban areas, other towns as small urban areas, and the countryside as rural areas. Elixhauser comorbidity indices (ECIs) were computed as proxies of patient health statuses based on diagnoses obtained from outpatient and inpatient records during the history period [32]. In addition, we considered whether patients had a diagnosis of diabetes or were being prescribed an antihyperlipidemic agent at the index dates and collected baseline data about health services utilization, and costs during the exposure period regardless of diagnoses. Antihyperlipidemic agents include medications listed in the WHO ATC Index under C10, and the KNHI codes for each drug used in the analysis are provided in the supporting information (S2 File).

### Statistical analysis

Descriptive analyses were conducted to evaluate the characteristics of patients in the four groups. Inter-group comparisons of continuous and categorical variables were performed using the Kruskal-Wallis test and the chi-squared test, respectively. Additionally, continuous variables in the four groups were analyzed in pairs using the Kruskal-Wallis test and Bonferroni test. Adjusted hazard ratios (aHRs) and 95% confidence intervals (CIs) for incidence of ASCVD in the four groups were estimated using a Cox proportional hazard regression model with adjustment for baseline characteristics (age, sex, insurance contribution group, living area, ECI, and antihyperlipidemic agent use). The Kaplan-Meier curves were presented and the log-rank test was performed for comparison incidences across the four groups. Odds ratios (ORs) for inappropriate medication adherence were estimated by logistic regression, and analyses were adjusted for baseline characteristics (age, sex, insurance contribution group, living area, and ECI). In cases of individuals whose information about insurance contribution and living area were missing, missing values were considered an independent category in statistical regression models. Sensitivity analyses were conducted in two ways to reassess the definition of the initial 3-year continuity as exposure. First, the outcome period was reduced to 5 years to investigate whether the impact changes as the outcome period decreases. Second, a time-dependent Cox model was employed, treating COCI as a time-dependent variable. Cumulative COCI for each individual was recomputed annually for the analysis, leading to a change in the groups to which included patients belonged each year. The analysis was conducted using SAS Ver. 9.4 (SAS Institute Inc, Cary, NC, USA), and statistical significance was accepted for *p* values <0.05.

## Results

### Baseline characteristics of the study population

**Fig 1:**
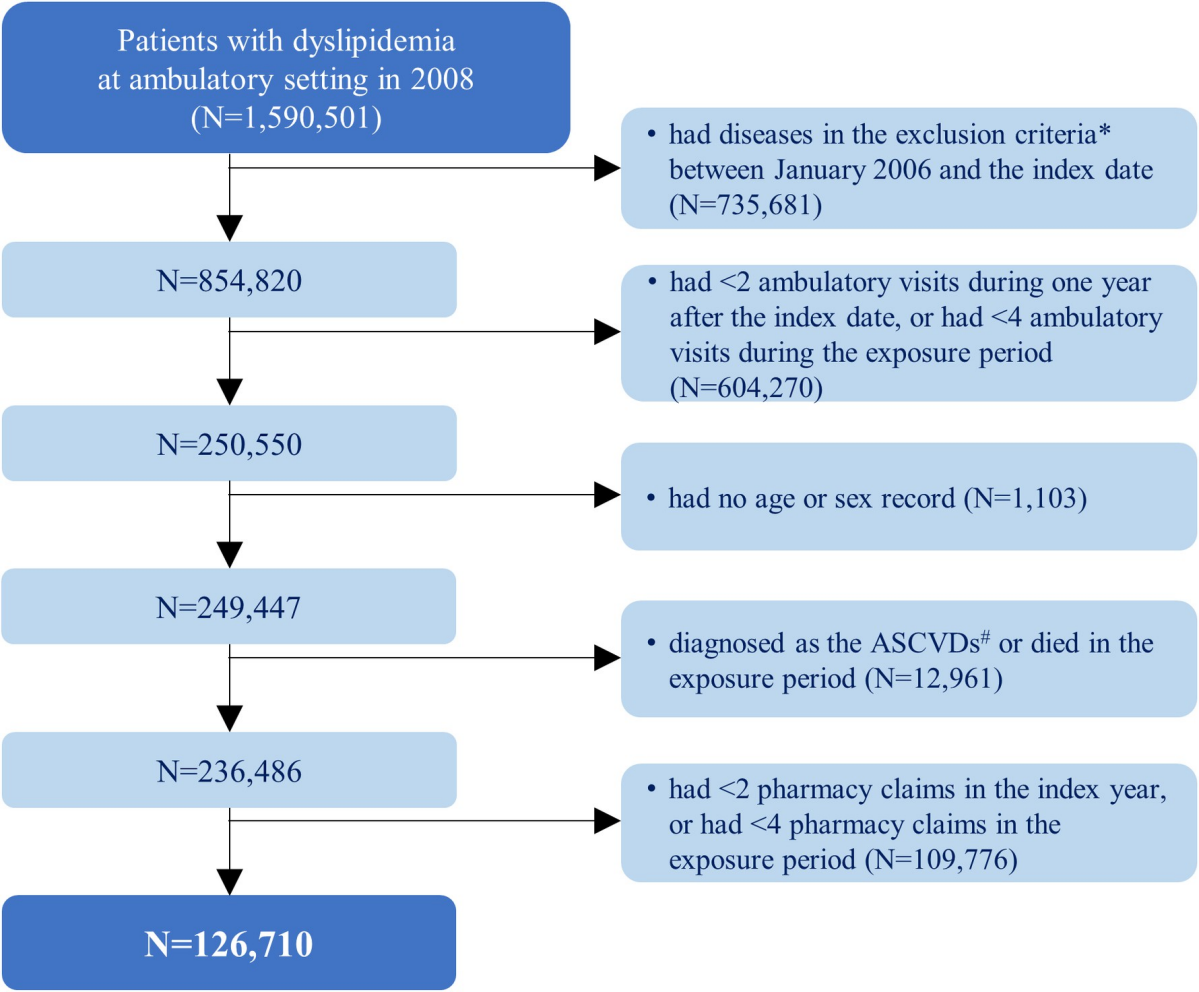
Selection of the study population. *The exclusion criteria included ischemic heart diseases, cerebrovascular diseases and related syndromes, diabetes mellitus with circulatory complications, and cancer. #ASCVD represents atherosclerotic cardiovascular disease and defines one of myocardial infarction, stable or unstable angina, ischemic stroke, and transient ischemic attack in this study.

The selection process is shown in Fig 1. A total of 126,710 patients were eligible for analysis.

The baseline characteristics of the study population are presented in Table 1. The study population had a median age of 52 years (interquartile range: 46–60); 14% were aged 65 or older. In this group, 56% were female, 45% came from households with high insurance contributions, and 49% resided in large urban areas. Notably, 4% were MedAid beneficiaries, exceeding the general population’s average of approximately 3%, and 16% had diabetes mellitus (DM). Among the study patients, 45% had an ECI score of zero, and a similar proportion (49%) were prescribed antihyperlipidemic agents at the index dates.

**Table 1:** Baseline characteristics of study population.

Variable		All patients (n = 126,710)	H^M^/H^P^ (n = 44,678)	H^M^/L^P^ (n = 24,270)	L^M^/H^P^ (n = 14,733)	L^M^/L^P^ (n = 43,029)	*p-value*
*General characteristic*, n (%)							
Sex	Women	70,363 (56)	23,170 (52)	12,789 (53)	8,727 (59)	25,677 (60)	<0.001
Age	median (IQR)	52 (46–60)	52 (45–59)	52 (46–59)	53 (46–60)	53 (46–60)	<0.001
	≥ 65 years	17,996 (14)	6,414 (14)	3,195 (13)	2,426 (16)	5,961 (14)	<0.001
Insurance contributions^a^	High	57,355 (45)	19,854 (44)	11,599 (48)	6,328 (43)	19,574 (45)	<0.001
	Moderate	36,528 (29)	13,188 (30)	6,863 (28)	4,177 (28)	12,300 (29)	
	Low	25,868 (20)	9,041 (20)	4,762 (20)	3,049 (21)	9,016 (21)	
Insurance program	NHI	121,409 (96)	42,629 (95)	23,518 (97)	13,749 (93)	41,513 (96)	<0.001
	MedAid	5,301 (4)	2,049 (5)	752 (3)	984 (7)	1,516 (4)	
Urbanization level of residence^b^	Large urban	62,503 (49)	22,186 (50)	12,129 (50)	7,136 (48)	21,052 (49)	<0.001
	Small urban	52,071 (41)	18,401 (41)	10,078 (42)	5,955 (40)	17,637 (41)	
	Rural	12,013 (9)	4,055 (9)	2,039 (8)	1,630 (11)	4,289 (10)	
Elixhauser comorbidity index	median (IQR)	1 (0–2)	1 (0–2)	1 (0–1)	1 (0–2)	1 (0–1)	<0.001
	0	57,246 (45)	19,953 (45)	11,448 (47)	6,218 (42)	19,627 (46)	<0.001
	1	37,338 (29)	13,061 (29)	7,055 (29)	4,469 (30)	12,753 (30)	
	2	19,542 (15)	7,157 (16)	3,564 (15)	2,389 (16)	6,432 (15)	
	3+	12,584 (10)	4,507 (10)	2,203 (9)	1,657 (11)	4,217 (10)	
Comorbidity- diabetes mellitus	Yes	20,155 (16)	8,220 (18)	4,140 (17)	2,021 (14)	5,774 (13)	<0.001
	No	106,555 (84)	36,458 (82)	20,130 (83)	12,712 (86)	37,255 (87)	
Antihyperlipidemic agent use	Yes	61,897 (49)	22,009 (49)	11,639 (48)	6,829 (46)	21,420 (50)	<0.001
	No	64,813 (51)	22,669 (51)	12,631 (52)	7,904 (54)	21,609 (50)	
*Annual health services utilization for all diseases during exposure period*, median (IQR)							
Ambulatory visits		18.3 (12.0–28.7)	17.7 (11.7–27.0)	17.0 (11.0–26.3)	20.7 (13.3–32.7)	19.3 (12.3–30.3)	<0.001
Pharmacist visits		15.3 (10.0–22.7)	15.0 (10.0–22.0)	14.3 (9.3–21.0)	16.7 (11.0–25.0)	15.7 (10.3–23.7)	<0.001
All medical costs (1,000 KRW^c^)	Total	1,277 (799–2,022)	1,235 (766–1,920)	1,287 (819–1,972)	1,324 (818–2,160)	1,304 (812–2,119)	<0.001
	Out-of-pocket payment	373 (223–589)	354 (207–554)	391 (240–601)	365 (214–593)	387 (234–617)	<0.001
	Public expenditure	875 (542–1,417)	853 (526–1,351)	870 (549–1,354)	919 (561–1,558)	891 (548–1,482)	<0.001

Abbreviations: COC = continuity of care; IQR = interquartile range; MedAid = Medical Aid; NHI = National Health Insurance.

*Note*: The group definitions are; H^M^ = high COC with doctor; L^M^ = low COC with doctor; H^P^ = high COC with pharmacist; and L^P^ = low COC with pharmacist. The *p-values* were calculated by the Kruskal-Wallis tests for continuous variables and by the chi-squared tests for categorical variables.

^a^Information for insurance contributions: Missing 6,959 (5.49%), 2,595 (5.81%), 1,046 (4.31%), 1,179 (8.00%), 2,139 (4.97%), respectively.

^b^Information for urbanization level of residence: Missing 123 (0.10%), 36 (0.08%), 24 (0.10%), 12 (0.08%), 51 (0.12%), respectively.

^c^1 US dollar = 1,200 KRW in Feb 2022.

The study patients were distributed across four groups as follows: H^M^/H^P^ 35% (n = 44,678), H^M^/L^P^ 19% (n = 24,270), L^M^/H^P^ 12% (n = 14,733), and LM/LP 34% (n = 43,029). The H^M^/H^P^ and H^M^/L^P^ groups had a higher representation of men (by 34%) and DM patients (by 13%) than the total cohort. Conversely, the L^M^/H^P^ and L^M^/L^P^ groups had more women (by 4%) and were slightly older with a reduced prevalence of DM (by 2~3%). Patients in the L^M^/H^P^ and L^M^/L^P^ groups frequented healthcare providers more often. Moreover, those in the L^M^/H^P^ group had lower out-of-pocket expenses but made greater use of public resources. In the H^M^/L^P^ group, 48% paid ’high’ insurance contributions, and 47% had an ECI score of zero, distinguishing them from other groups. The MedAid beneficiary rate in this group (3%) matched the national average.

The L^M^/H^P^ group stood out with the highest percentage of elderly patients (16% aged ≥65 years) and MedAid beneficiaries (7%). A smaller portion were affluent (43% with ’high’ insurance contributions), more lived in rural areas (11%), and they had a higher likelihood of an ECI score of ≥3 (11%) but a lower likelihood of DM (14%).

### Changes in continuity of care

Throughout the exposure period, the average COCIs for doctor visits and pharmacist visits among the study patients were 0.86 and 0.74, respectively. These figures declined to 0.58 and 0.51, respectively, during the ten-year study duration (S2 Table). Specifically, the COCI in the H^M^/H^P^ group declined from 1.0 to 0.81 for both providers. In the H^M^/L^P^ group, it decreased from 1.0 to 0.78 for doctors and from 0.50 to 0.42 for pharmacists. The L^M^/H^P^ group saw a decrease from 0.59 to 0.47 for doctors and from 1.0 to 0.71 for pharmacists. Meanwhile, the L^M^/L^P^ group experienced a decrease from 0.48 to 0.43 for doctors and from 0.44 to 0.38 for pharmacists.

### Incidence of atherosclerotic cardiovascular disease

**Fig 2:**
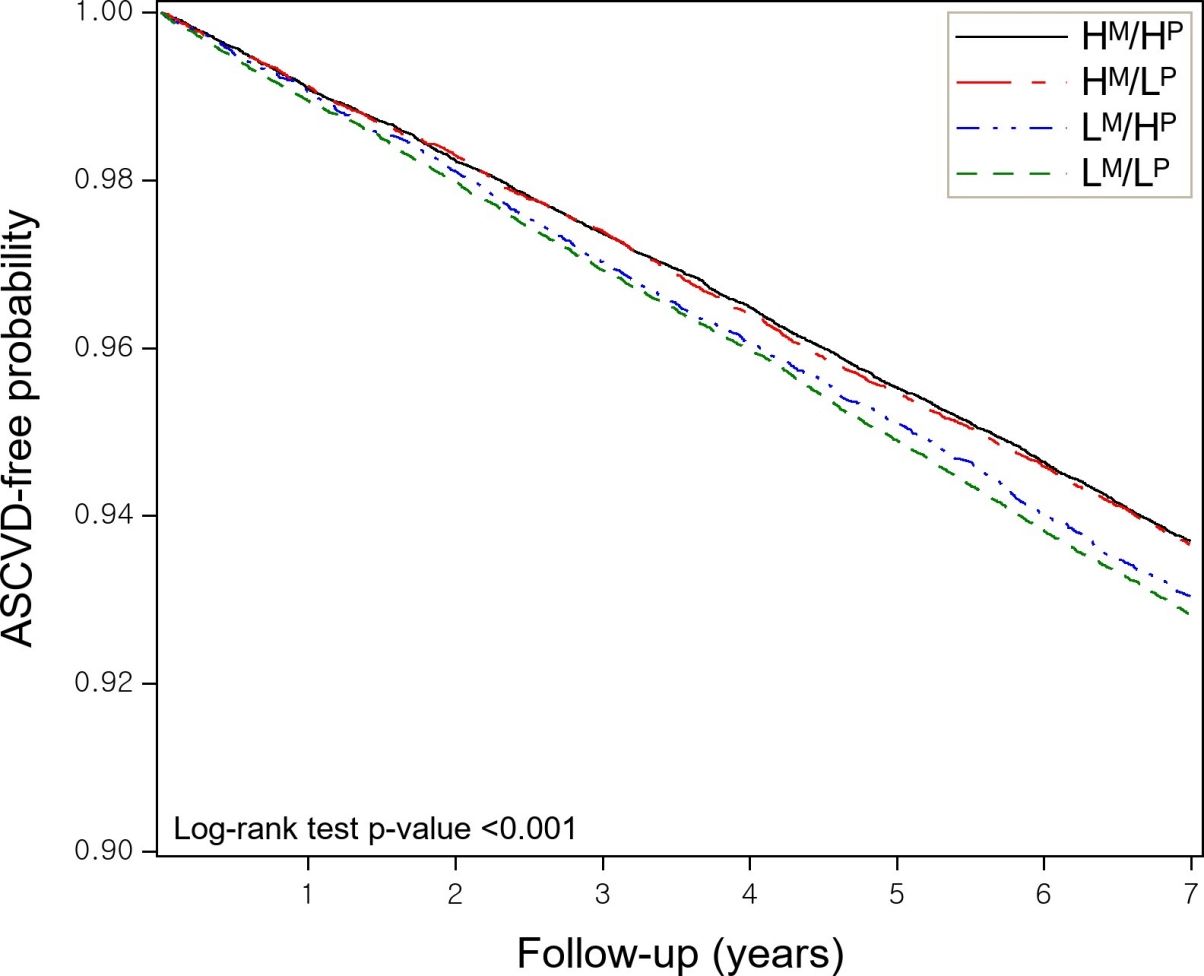
ASCVD-free probability by level of continuity of care. ASCVD = atherosclerotic cardiovascular disease. The group definitions are; H^M^ = high COC with doctor; L^M^ = low COC with doctor; H^P^ = high COC with pharmacist; and L^P^ = low COC with pharmacist.

8,337 patients (7%) developed an ASCVD during the seven-year outcome period (Table 2). Corresponding percentages of ASCVD development in the H^M^/H^P^, H^M^/L^P^, L^M^/H^P^, and L^M^/L^P^ groups were 6.2%, 6.3%, 6.8%, and 7.1%, respectively. After adjusting for confounding covariates, only the L^M^/L^P^ group had a significantly higher risk of ASCVD than the H^M^/H^P^ group (the reference group) (aHR = 1.16 [95% confidence interval = 1.10~1.22]). The probabilities of patients not developing an ASCVD during the seven-year outcome period, as determined by primary analysis are provided in Fig 2. In a sensitivity analysis where the outcome period was restricted to five years, it was revealed that L^M^/L^P^ group exhibited a similar level of ASCVD risk (aHR = 1.17 [1.10~1.24]). Another sensitivity analysis using a time-dependent model over seven-year outcome period, yielding an aHR of 1.27 [1.20–1.38], which was consistent with the primary analysis findings.

**Table 2:** Risks of occurrence of ASCVD in Korean dyslipidemia patients by level of continuity of care.

Outcome	H^M^/H^P^ (n = 44,678)	H^M^/L^P^ (n = 24,270)	L^M^/H^P^ (n = 14,733)	L^M^/L^P^ (n = 43,029)
No. of patients newly developed ASCVD (%)	2,772 (6.2)	1,516 (6.3)	1,005 (6.8)	3,044 (7.1)
*Cox’s hazards regression model*				
Unadjusted HR (95% CI)	1.00 (Reference)	1.01 (0.95–1.07)	1.11 (1.03–1.19)^†^	1.14 (1.09–1.20)^§^
Adjusted HR (95% CI)	1.00 (Reference)	1.03 (0.96–1.09)	1.06 (0.98–1.14)	1.16 (1.10–1.22)^§^
*Cox’s hazards regression model for 5-year outcome period (sensitivity analysis 1)*				
Unadjusted HR (95% CI)	1.00 (Reference)	1.01 (0.94–1.09)	1.10 (1.01–1.20)*	1.14 (1.08–1.22)^§^
Adjusted HR (95% CI)	1.00 (Reference)	1.03 (0.96–1.11)	1.05 (0.96–1.14)	1.17 (1.10–1.24)^§^
*Cox’s hazards regression model for time dependent COCI for 7-year outcome period (sensitivity analysis 2)*				
Unadjusted HR (95% CI)	1.00 (Reference)	1.03 (0.96–1.11)	1.23 (1.13–1.32)^§^	1.24 (1.17–1.31)^§^
Adjusted HR (95% CI)	1.00 (Reference)	1.04 (0.97–1.12)	1.16 (1.08–1.26)^†^	1.27 (1.20–1.38)^§^

Abbreviations: ASCVD = atherosclerotic cardiovascular disease; CI = confidential interval; HR = hazard ratio.

*Note*: The group definitions are; H^M^ = high COC with doctor; L^M^ = low COC with doctor; H^P^ = high COC with pharmacist; and L^P^ = low COC with pharmacist. The adjusted HR was analyzed after adjusting for covariates including sex, age, insurance contribution, urbanization level of residence, Elixhauser comorbidity index, and antihyperlipidemic agent use.

*p < 0.05

^†^*p* < 0.01

^§^*p* < 0.001.

### Health services utilization, disease-related costs, and adherence to medication

Analysis results for secondary outcomes are displayed in Table 3. The number of patients hospitalized due to ASCVD during the outcome period were significantly higher in the L^M^/H^P^ and L^M^/L^P^ groups (by 5%~6%) than in the H^M^/H^P^ group. The L^M^/L^P^ group exhibited a shorter hospital stay compared to the other three groups. The number of patients visiting EDs was considerably greater in the L^M^/H^P^ group (by 5%) than in the H^M^/H^P^ group.

**Table 3:** Health services utilization, costs and medication adherence by level of continuity of care.

Outcome	H^M^/H^P^ (n = 44,678)	H^M^/L^P^ (n = 24,270)	L^M^/H^P^ (n = 14,733)	L^M^/L^P^ (n = 43,029)	*p-value*
*Health services utilization due to ASCVD for 7-year outcome period*					
No. of patients hospitalized (%)	2,100 (4.7)	1,132 (4.7)	765 (5.2)	2,257 (5.3)	<0.001
Average length of hospitalization days per patient per year, median (IQR)	10 (4–24)	10 (4–25)	10 (4–26)	9 (3–23)^a^	0.014
No. of patients visiting ED (%)	1,145 (2.6)	618 (2.6)	445 (3.0)	1,170 (2.7)	0.013
Frequency of visiting ED per patient per year, median (IQR)	1 (1–1)	1 (1–1)	1 (1–1)	1 (1–1)	0.767
*Disease-related medical costs*^b^ *for 7-year outcome period*					
Overall disease related medical costs 1,000 KRW per patient per year, median (IQR)	116 (41–245)	125 (52–257)^a^	111 (37–254)	129 (56–274)^a^	<0.001
Public expenditure	79 (26–171)	81 (32–174)^a^	76 (24–179)	87 (37–192)^a^	<0.001
Out-of-pocket payment	31 (10–71)	36 (14–82)^a^	28 (8–67)^a^	36 (14–80)^a^	<0.001
*Inappropriate medication adherence*^*c*^ *for 10-year study period*					
Unadjusted OR (95% CI)	1.0 (Reference)	1.02 (0.99–1.05)^§^	1.62 (1.56–1.69)^§^	2.22 (2.15–2.29)^§^	-
Adjusted OR (95% CI)	1.0 (Reference)	1.03 (0.99–1.06)^§^	1.67 (1.60–1.74)^§^	2.26 (2.19–2.34)^§^	-

Abbreviations: ASCVD = atherosclerotic cardiovascular disease; CI = confidential interval; ED = emergency department; IQR = interquartile range; OR = odds ratio.

*Note*: The group definitions are; H^M^ = high COC with doctor; L^M^ = low COC with doctor; H^P^ = high COC with pharmacist; and L^P^ = low COC with pharmacist. The *p-values* were calculated by the Kruskal-Wallis tests for continuous variables and by the chi-squared tests for categorical variables. The adjusted OR was analyzed after adjusting for covariates including sex, age, insurance contribution, urbanization level of residence, and Elixhauser comorbidity index.

^a^Statistically significant changes were seen compared to the H^M^/H^P^ group. Continuous variables in the four groups were analyzed in pairs using the Kruskal-Wallis and Bonferroni’s tests.

^b^Disease-related medical costs included costs for treating dyslipidemia and ASCVD; 1 US dollar = 1,200 KRW in Feb 2022.

^c^Medication Possession Ratio of < 0.8 was defined as inappropriate medication adherence.

^§^*p* < 0.001.

Disease-related medical costs were significantly higher in the H^M^/L^P^ and L^M^/L^P^ groups by 8~11% for overall expenditure, by 3~10% for public expenditure, and by 16% for out-of-pocket expenditure than in the H^M^/H^P^ group. Contrastingly, out-of-pocket paid in the L^M^/H^P^ group was significantly lower (by about 10%) than in the H^M^/H^P^ group. Overall and public expenditures in the L^M^/H^P^ group were not different from those of the H^M^/H^P^. Results from the post-hoc tests are reported in S3 Table.

After adjusting for covariates, risks of inappropriate medication adherence were 1.03 times higher in the H^M^/L^P^, 1.67 in the L^M^/H^P^, and 2.26 in the L^M^/L^P^ compared to the H^M^/H^P^ group. More detailed analysis results regarding medication adherence are provided in S4 Table.

## Discussion

We examined nationwide claims data to assess how the level of patient relational COC with doctors and community pharmacists influences the clinical and economic outcomes of patients with dyslipidemia. Patients who had low levels of relational COC with their doctors and pharmacists were 1.16 times more likely to develop ASCVD, had higher healthcare service utilization, and accrued increased costs.

Clearly, the group having high-level COCs for both providers had a significantly lower risk of ASCVD than the group with low-level COC for both. The two mixed groups who had high-level COCs either for doctors or for pharmacists had intermediate risks, although these were not significantly different from that of the group with high continuity for both providers. The sequence of risk growth is interesting. Risks of ASCVD increased in the order H^M^/H^P^, H^M^/L^P^, L^M^/H^P^, and L^M^/L^P^. The same sequence order was observed for the risk of inappropriate medication adherence. Study patients with high COC only for their doctors (H^M^/L^P^) had higher medication adherence than those with high COC only for their pharmacists (L^M^/H^P^), which might be because antihyperlipidemic agents are prescription-only-medicines in South Korea. Although a recent systematic review suggested that maintaining a high level of continuity with pharmacists could be linked to better medication adherence [21], it’s important to note that medication adherence can only be achieved with a prescription from a doctor. Unlike the occurrences of ASCVD or adherence to medications, the costs related to the disease were minimal among groups with a high level of COC with pharmacists (H^M^/H^P^ and L^M^/H^P^). However, this should be cautiously interpreted as the data suggests statistical significance but may not have a direct connection to clinical significance. Further studies on this topic are required. In the L^M^/L^P^ group, a notable observation is that the hospital stay due to ASCVD is statistically significantly shorter than in the other three groups; however, the clinical significance remains uncertain when comparing absolute values.

Characteristics of the L^M^/H^P^ (high COC only for pharmacists) are worth noting. Patients in this group were older, economically disadvantaged, lived in rural areas, seemed to have low disease severities, and spent least which was attributed to a significantly low out-of-pocket spending. However, they had the highest emergency department visit rate. Study results suggest that relational COC with pharmacist is less effective than that with doctor in terms of clinical outcomes, but pharmacist services were associated with a reduction in overall costs. These also imply that there may be an overlooked group of patients who, due to financial constraints, limited accessibility, or misconceptions about their illness, depend more on the relationship with pharmacists. Conventionally, pharmacists are regarded as professionals who have a significant role in addressing these gaps [33], which is supported by findings of the study. Furthermore, this study highlighted the importance of pharmacists needing to devise strategies aimed at enhancing clinical outcomes by optimizing the timing of referrals for patients who rely more on having a good relationship with pharmacists.

To the best of our knowledge, this is the first study to investigate the impacts of COC between patients and two types of healthcare providers in a large-scale cohort of dyslipidemia patients. The study relied on the examination of nationwide claims data, and the primary analysis along with two distinct sensitivity analyses generated uniform findings, indicating the robust reliability and validity of the study.

However, several limitations should be considered when interpreting our results. First, the analysis relied on claims data, which restricted our ability to include crucial factors such as laboratory results and individual lifestyles (e.g., smoking, exercise intensity, and obesity). This limitation prompted us to adopt a conservative definition of ASCVD, potentially resulting in an underestimation of its incidence and the inter-group hazard ratio differences. Second, we were unable to evaluate the extent of individual attributes in the relationships between patients and their doctors or pharmacists, which could be closely tied to patients’ decisions regarding future visits. Furthermore, we were also unable to address the effects of third parties, such as nurses and caregivers, on the relationships. Third, although we assessed relationships between patients and doctors or pharmacists concurrently, providers did not establish shared care plans for patients. Fourth, this study included the patients with newly developed dyslipidemia and observed the prognosis of illness for 10 years, which limits the generalizability of the study findings to those in other dyslipidemic conditions.

## Conclusions

Dyslipidemia patients who maintain a strong relational care continuity with their doctors and pharmacists attain better clinical outcomes. Patients who had high levels of COC with their doctors and pharmacists had a reduced likelihood of experiencing ASCVD, utilized healthcare services less frequently, and spent lower costs. The two mixed groups who had high-level COCs with either doctors or pharmacists exhibited intermediate risks. COC with pharmacist is less effective than COC with doctor when it comes to clinical results, but it was observed that pharmacist services led to a decrease in overall expenses. Careful attention is needed for patients with high care continuity only with pharmacists to ascertain whether they are reducing doctor visits due to financial constraints, limited accessibility, or misconceptions about their illness.

## Supporting information

S1 Fig Defined study periods. (TIF)

S2 Fig Distribution of COCI by provider. (TIF)

S1 Table ICD-10 codes used for data analysis. (DOCX)

S2 Table Changes in continuity of care index by continuity group. (DOCX)

S3 Table Results from the post-hoc tests of inter-group comparison in costs. (DOCX)

S4 Table Detailed analysis results regarding MPR. (DOCX)

S1 File Bice & Boxerman continuity of care index. (DOCX)

S2 File List of antihyperlipidemics. (XLSX)
