# Supplementary figures and images for: Continuity of care between dyslipidemia patients and multiple providers: A cohort study
Source: PLoS One. 2024 May 2;19(5):e0300745. doi: 10.1371/journal.pone.0300745 (PMC11065238; doi:10.1371/journal.pone.0300745)

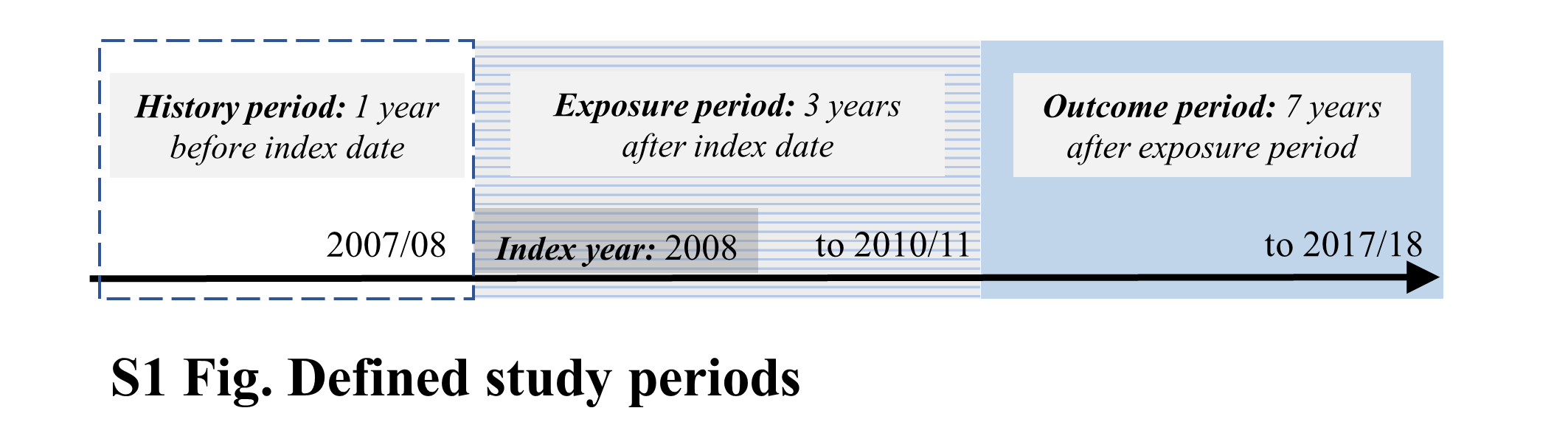

Supplement: S1 Fig — (TIF) [file pone.0300745.s001.tif]

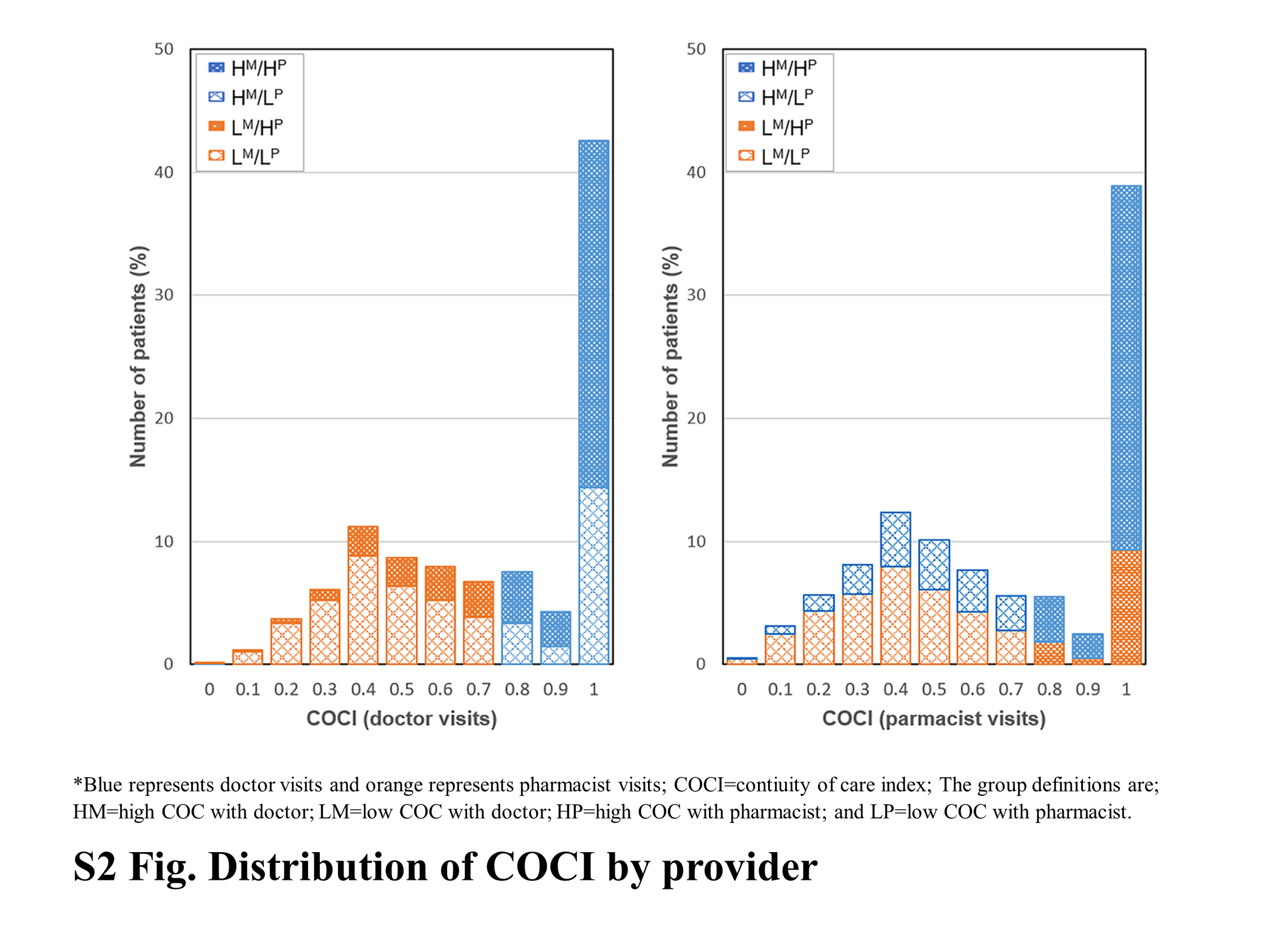

Supplement: S2 Fig — (TIF) [file pone.0300745.s002.tif]
