# Supplementary material for: Continuity of care between dyslipidemia patients and multiple providers: A cohort study
Source: PLoS One. 2024 May 2;19(5):e0300745. doi: 10.1371/journal.pone.0300745 (PMC11065238; doi:10.1371/journal.pone.0300745)
Supplement: S1 Table — (DOCX) [file pone.0300745.s003.docx]

**Supporting Information**

**S1 Table. ICD-10 codes used for data analysis**

| **Diagnosis** | **Code** |
| --- | --- |
| Cancer | C00 to C97 |
| Cerebrovascular disease or a related syndrome | I60 to I69, G45 to G46 |
| Diabetes mellitus | E10.0-3, E10.6-9, E11.0-4, E11.6-9, E12.0-4, E12.6-9, E13.0-4, E13.6-9, E14.0-4, E14.6-9 |
| Diabetes mellitus with circulatory complications | E10.5, E11.5, E12.5, E13.5, E14.5 |
| Dyslipidemia | E78.0 to E78.9 |
| Hypertensive disease | I10 to I13, I15 |
| Ischemic heart disease | I20 to I25 |
| Ischemic stroke | I63.0-6, I63.8-9, |
| Myocardial infarction | I21.0-4, I21.9, I22.0-1, I22.8-9 |
| Stable or unstable angina | I20 |
| Transient ischemic attack | G45.0-3, G45.8-9 |
| ASCVD |  |
| Ischemic stroke | I63.0-6, I63.8-9 |
| Myocardial infarction | I21.0-4, I21.9, I22.0-1, I22.8-9 |
| Stable or unstable angina | I20 |
| Transient ischemic attack | G45.0-3, G45.8-9 |
| *Assessment* |  |
| CT scan or MRI | HA451, HA461, HA471, HA441, HE101, HE201, HE401, HE501, HE102, HE202, HE402, HE502 |
| *Treatment* |  |
| CABG or PCI | HA670, HA680, HA681, HA682, M6551, M6552, M6561, M6562, M6563, M6564, M6571, M6572, M6633, M6634, O1641, O1642, O1647, OA641, OA642, OA647 |

Abbreviations: ASCVD= atherosclerotic cardiovascular disease; CABG= coronary artery bypass graft surgery; CT=computed tomography; ICD-10=International Classification of Disease 10th revision; MRI=magnetic resonance imaging; PCI=percutaneous coronary intervention
