# Supplementary material for: Continuity of care between dyslipidemia patients and multiple providers: A cohort study
Source: PLoS One. 2024 May 2;19(5):e0300745. doi: 10.1371/journal.pone.0300745 (PMC11065238; doi:10.1371/journal.pone.0300745)
Supplement: S2 Table — (DOCX) [file pone.0300745.s004.docx]

**Supporting Information**

**S2 Table. Changes in Continuity of Care Index by continuity group**

|  | | **H^M^/H^P^**  **(n=44,678)** | **H^M^/L^P^**  **(n=24,270)** | **L^M^/H^P^**  **(n=14,733)** | **L^M^/L^P^**  **(n=43,029)** | ***p-value*** |
| --- | --- | --- | --- | --- | --- | --- |
| *Exposure period (3 years)* | | | | | | |
| Doctor visit  median (IQR) | COCI overall | 0.86 (0.52-1.00) | | | | - |
|  | COCI by group | 1.00 (1.00-1.00) | 1.00 (1.00-1.00) | 0.59 (0.47-0.70) | 0.48 (0.38-0.60) | <0.001 |
|  | No. of visits | 16 (9-29) | 16 (10-26) | 14 (8-24) | 15 (9-22) | <0.001 |
| Pharmacist visit  median (IQR) | COCI overall | 0.74 (0.47-1.00) | | | | - |
|  | COCI by group | 1.00 (1.00-1.00) | 0.50 (0.40-0.63) | 1.00 (1.00-1.00) | 0.44 (0.32-0.56) | <0.001 |
|  | No. of visits | 13 (7-24) | 13 (7-22) | 10 (5-18) | 11 (7-18) | <0.001 |
| *Exposure plus outcome period (10 years)* | | | | | | |
| Doctor visit  median (IQR) | COCI overall | 0.58 (0.40-0.88) | | | | - |
|  | COCI by group | 0.81 (0.51-1.00) | 0.78 (0.50-1.00) | 0.47 (0.34-0.64) | 0.43 (0.30-0.62) | <0.001 |
|  | No. of visits | 49 (23-83) | 49 (27-78) | 43 (20-74) | 48 (27-74) | <0.001 |
| Pharmacist visit  median (IQR) | COCI overall | 0.51 (0.34-0.82) | | | | - |
|  | COCI by group | 0.81 (0.49-1.00) | 0.42 (0.30-0.59) | 0.71 (0.47-1.00) | 0.38 (0.26-0.54) | <0.001 |
|  | No. of visits | 35 (14-64) | 35 (17-60) | 27 (11-53) | 33 (17-55) | <0.001 |

Abbreviations: COC=continuity of care; COCI=continuity of care index; IQR=interquartile range; H^M^=high COC with doctor; L^M^=low COC with doctor; H^P^=high COC with pharmacist; L^P^=low COC with pharmacist.

*Note*: The group definitions are; H^M^=high COC with doctor; L^M^=low COC with doctor; H^P^=high COC with pharmacist; and L^P^=low COC with pharmacist. The *p-values* were calculated by the Kruskal-Wallis tests.
