# Supplementary material for: Continuity of care between dyslipidemia patients and multiple providers: A cohort study
Source: PLoS One. 2024 May 2;19(5):e0300745. doi: 10.1371/journal.pone.0300745 (PMC11065238; doi:10.1371/journal.pone.0300745)
Supplement: S3 Table — (DOCX) [file pone.0300745.s005.docx]

**Supporting Information**

**S3 Table. Results from the post-hoc tests of inter-group comparison in costs**

a) Overall disease-related medical costs

| ***p-value*** | **H^M^/H^P^** | **H^M^/L^P^** | **L^M^/H^P^** | **L^M^/L^P^** |
| --- | --- | --- | --- | --- |
| **H^M^/H^P^** |  | <0.0001 | 0.5772 | <0.0001 |
| **H^M^/L^P^** | <0.0001 |  | <0.0001 | <0.0001 |
| **L^M^/H^P^** | 0.5772 | <0.0001 |  | <0.0001 |
| **L^M^/L^P^** | <0.0001 | <0.0001 | <0.0001 |  |

*Note*: The group definitions are; H^M^=high COC with doctor; L^M^=low COC with doctor; H^P^=high COC with pharmacist; and L^P^=low COC with pharmacist. Continuous variables in the four groups were analyzed in pairs using the Bonferroni’s tests.

b) Public expenditure

| ***p-value*** | **H^M^/H^P^** | **H^M^/L^P^** | **L^M^/H^P^** | **L^M^/L^P^** |
| --- | --- | --- | --- | --- |
| **H^M^/H^P^** |  | <0.0001 | 1.0000 | <0.0001 |
| **H^M^/L^P^** | <0.0001 |  | <0.0001 | <0.0001 |
| **L^M^/H^P^** | 1.0000 | <0.0001 |  | <0.0001 |
| **L^M^/L^P^** | <0.0001 | <0.0001 | <0.0001 |  |

*Note*: The group definitions are; H^M^=high COC with doctor; L^M^=low COC with doctor; H^P^=high COC with pharmacist; and L^P^=low COC with pharmacist. Continuous variables in the four groups were analyzed in pairs using the Bonferroni’s tests.

c) Out-of-pocket payment

| ***p-value*** | **H^M^/H^P^** | **H^M^/L^P^** | **L^M^/H^P^** | **L^M^/L^P^** |
| --- | --- | --- | --- | --- |
| **H^M^/H^P^** |  | <0.0001 | <0.0001 | <0.0001 |
| **H^M^/L^P^** | <0.0001 |  | <0.0001 | 1.0000 |
| **L^M^/H^P^** | <0.0001 | <0.0001 |  | <0.0001 |
| **L^M^/L^P^** | <0.0001 | 1.0000 | <0.0001 |  |

*Note*: The group definitions are; H^M^=high COC with doctor; L^M^=low COC with doctor; H^P^=high COC with pharmacist; and L^P^=low COC with pharmacist. Continuous variables in the four groups were analyzed in pairs using the Bonferroni’s tests.
