# Supplementary material for: Continuity of care between dyslipidemia patients and multiple providers: A cohort study
Source: PLoS One. 2024 May 2;19(5):e0300745. doi: 10.1371/journal.pone.0300745 (PMC11065238; doi:10.1371/journal.pone.0300745)
Supplement: S4 Table — (DOCX) [file pone.0300745.s006.docx]

**Supporting Information**

**S4 Table. Detailed analysis results regarding MPR**

a) Number of patients in MPR category by period

| **Period** | | **Overall** | **H^M^/H^P^** | **H^M^/L^P^** | **L^M^/H^P^** | **L^M^/L^P^** |
| --- | --- | --- | --- | --- | --- | --- |
| N (%) | | 126,710 (100.0) | 44,678 (100.0) | 24,270 (100.0) | 14,733 (100.0) | 43,029 (100.0) |
| Exposure period | 0≤MPR<0.8 | 77,397 (61.1) | 22,003 (49.2) | 13,264 (54.7) | 8,577 (58.2) | 33,553 (78.0) |
|  | 0.8≤MPR | 49,313 (38.9) | 22,675 (50.8) | 11,006 (45.3) | 6,156 (41.8) | 9,476 (22.0) |
| Exposure and 5-year outcome period | 0≤MPR<0.8 | 89,191 (70.4) | 28,124 (62.9) | 15,554 (64.1) | 10,734 (72.9) | 34,779 (80.8) |
|  | 0.8≤MPR | 37,519 (29.6) | 16,554 (37.1) | 8,716 (35.9) | 3,999 (27.1) | 8,250 (19.2) |
| Exposure and 7-year outcome period | 0≤MPR<0.8 | 90,843 (71.7) | 29,136 (65.2) | 15,925 (65.6) | 11,089 (75.3) | 34,693 (80.6) |
|  | 0.8≤MPR | 35,867 (28.3) | 15,542 (34.8) | 8,345 (34.4) | 3,644 (24.7) | 8,336 (19.4) |

Abbreviation: MPR= Medication Possession Ratio.

*Note*: The group definitions are; H^M^=high COC with doctor; L^M^=low COC with doctor; H^P^=high COC with pharmacist; and L^P^=low COC with pharmacist. Medication Possession Ratio of < 0.8 was defined as inappropriate medication adherence.

b) Risk of inappropriate medication adherence by period

| Adjusted OR (95% confidence interval) | Exposure period | Exposure and 5-year outcome period | Exposure and 7-year outcome period |
| --- | --- | --- | --- |
| **H^M^/H^P^** | Reference | Reference | Reference |
| **H^M^/L^P^** | 1.25 (1.22-1.29) | 1.06 (1.03-1.10) | 1.03 (0.99-1.06) |
| **L^M^/H^P^** | 1.46 (1.41-1.52) | 1.61 (1.55-1.68) | 1.67 (1.60-1.74) |
| **L^M^/L^P^** | 3.72 (3.61-3.84) | 2.53 (2.45-2.61) | 2.26 (2.19-2.34) |

*Note*: The group definitions are; H^M^=high COC with doctor; L^M^=low COC with doctor; H^P^=high COC with pharmacist; and L^P^=low COC with pharmacist.

The adjusted OR was analyzed after adjusting for covariates including sex, age, insurance contribution, urbanization level of residence, and Elixhauser comorbidity index.
