# Supplementary material for: Continuity of care between dyslipidemia patients and multiple providers: A cohort study
Source: PLoS One. 2024 May 2;19(5):e0300745. doi: 10.1371/journal.pone.0300745 (PMC11065238; doi:10.1371/journal.pone.0300745)
Supplement: S1 File — (DOCX) [file pone.0300745.s007.docx]

**Supporting Information**

**Bice & Boxerman continuity of care index**

Bice & Boxerman continuity of care index (COCI) was calculated using the following formula.

COCI = $\frac{\sum_{j=1}^{M} n_{j}^{2}-N}{N(N-1)}$

Where N is total number of visits made by a patient to a doctor or pharmacist, *n_j_* is number of patient visits with provider *j*, and *M* is number of visits made to the provider visited most. Patient COCIs range between zero and one, where one indicates the highest possible continuity with healthcare provider, and zero indicates the absence of continuity and the use of different providers for each visit.
